# Proteomic profiling of hydatid fluid from pulmonary cystic echinococcosis

**DOI:** 10.1186/s13071-022-05232-8

**Published:** 2022-03-21

**Authors:** Guilherme Brzoskowski dos Santos, Edileuza Danieli da Silva, Eduardo Shigueo Kitano, Maria Eduarda Battistella, Karina Mariante Monteiro, Jeferson Camargo de Lima, Henrique Bunselmeyer Ferreira, Solange Maria de Toledo Serrano, Arnaldo Zaha

**Affiliations:** 1 Laboratório de Biologia Molecular de Cestódeos, Centro de Biotecnologia, Universidade Federal do Rio Grande do Sul, Porto Alegre, Brazil; 2 Laboratório de Genômica Estrutural E Funcional, Centro de Biotecnologia, Universidade Federal do Rio Grande do Sul, Porto Alegre, Brazil; 3 Laboratório de Toxinologia Aplicada, Center of Toxins, Immune-Response and Cell Signaling (CeTICS), Instituto Butantan, São Paulo, Brazil

**Keywords:** *Echinococcus granulosus*, *Echinococcus ortleppi*, Secretome, Hydatid fluid, Parasite proteomics, Host-parasite interface

## Abstract

**Background:**

Most cystic echinococcosis cases in Southern Brazil are caused by *Echinococcus granulosus* and *Echinococcus ortleppi*. Proteomic studies of helminths have increased our knowledge about the molecular survival strategies that are used by parasites. Here, we surveyed the protein content of the hydatid fluid compartment in *E. granulosus* and *E. ortleppi* pulmonary bovine cysts to better describe and compare their molecular arsenal at the host-parasite interface.

**Methods:**

Hydatid fluid samples from three isolates of each species were analyzed using mass spectrometry-based proteomics (LC-MS/MS). In silico functional analyses of the identified proteins were performed to examine parasite survival strategies.

**Results:**

The identified hydatid fluid protein profiles showed a predominance of parasite proteins compared to host proteins that infiltrate the cysts. We identified 280 parasitic proteins from *E. granulosus* and 251 from *E. ortleppi*, including 52 parasitic proteins that were common to all hydatid fluid samples. The in silico functional analysis revealed important molecular functions and processes that are active in pulmonary cystic echinococcosis, such as adhesion, extracellular structures organization, development regulation, signaling transduction, and enzyme activity.

**Conclusions:**

The protein profiles described here provide evidence of important mechanisms related to basic cellular processes and functions that act at the host-parasite interface in cystic echinococcosis. The molecular tools used by *E. granulosus* and *E. ortleppi* for survival within the host are potential targets for new therapeutic approaches to treat cystic echinococcosis and other larval cestodiases.

**Graphical Abstract:**

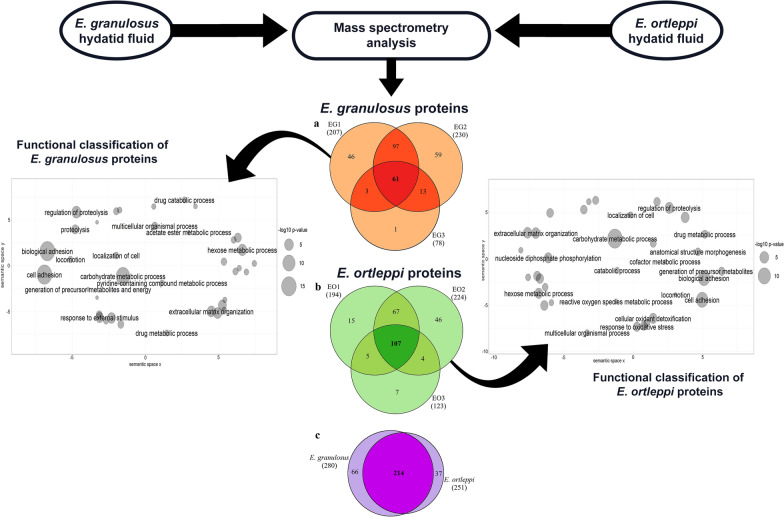

**Supplementary Information:**

The online version contains supplementary material available at 10.1186/s13071-022-05232-8.

## Background

Echinococcosis is caused by infection with flatworms from the genus *Echinococcus*. Depending on the species causing the infection, distinct morphological features can be observed because of differences in larval stage development [1]. Presently, the *E. granulosus *sensu lato (s.l.) complex is formed by five species: *Echinococcus granulosus *sensu stricto (s.s.; G1, G2 and G3), *Echinococcus equinus* (G4) *Echinococcus ortleppi* (G5), *Echinococcus canadensis* (G6-G8, G10), and *Echinococcus felidis* [2–5]. *Echinococcus granulosus* (s.s.) (G1, sheep strain) and *E. ortleppi* (G5, cattle strain) are etiological agents of cystic echinococcosis, which is characterized by the growth of the parasite’s larval stage (metacestode) as an unilocular, fluid-filled cyst (the hydatid cyst) in the viscera of suitable intermediate hosts (mainly cattle and sheep). Humans can be accidental hosts and develop cystic echinococcosis [3, 6]. In terms of epidemiology, *E. granulosus* (s.s.) is the most relevant species due to its worldwide occurrence, with high prevalence in domestic animals and humans [7]. *Echinococcus ortleppi* seems to be well adapted to cattle, although other intermediate hosts, including humans, can also be infected by this species [7, 8]. *Echinococcus ortleppi ortleppi* differs markedly in both larval and adult morphology from that of *E. granulosus* (s.s.), presenting a short development time in dogs [6, 9].

In *E. granulosus* and *E. ortleppi* life cycles [10], intermediate hosts become infected upon ingestion of parasite eggs. Egg hatching releases oncospheres, which develop into hydatid cysts in the host viscera (mainly liver and lungs). The hydatid cyst wall is formed by an external acellular, mucin-based laminated layer and an internal germinative layer. The germinative layer gives rise to brood capsules, where pre-adults (protoscoleces; PSCs) are produced by asexual reproduction. When PSCs are ingested by definitive hosts (canids, such as domestic dogs or wolves), they mature into adult worms within the small intestine, where they produce eggs that are released into the environment with host feces.

The hydatid cyst causes a chronic infection because it can survive and grow for decades in the host, in most cases remaining fertile, with full capacity to generate PSCs [4]. To achieve this, the parasites adopt a wide repertoire of molecular strategies to evade host defense mechanisms and acquire nutrients necessary for their development [11]. Such strategies allow parasite survival and development despite chronic exposure to a hostile environment created by the host response against infection. The liquid that fills the hydatid cyst, the hydatid fluid (HF), contains parasite excretory-secretory (ES) products and host proteins, making it a good component from which to analyze relevant molecules [12–14]. Although the HF is an inner component of the metacestode, it contains proteins that interact with the host. This can be evidenced by the humoral response to HF antigens detected in the host serum [11, 15]. Also, the germinative layer has secretory activity in its outer surface, since the presence of 14-3-3 and enolase in the laminated layer has already been observed [16, 17]. Recently, *E. granulosus* exosomes were detected in serum from patients with cystic echinococcosis [18], and the interaction of extracellular vesicles produced by *Echinococcus* with mammalian cells have been demonstrated in vitro [19]. Extracellular vesicles are carriers for different biomolecules and could act in the transfer of proteins through the hydatid cyst wall.

Despite its preference for ovine hosts, *E. granulosus* can also successfully infect, grow, and asexually reproduce in bovine hosts, although with less efficiency than *E. ortleppi* [4, 20]. For bovine hosts, the *E. ortleppi* cyst fertility rate is high (> 90%), while for *E. granulosus*, it normally does not exceed 30% [6, 21–23]. Moreover, *E. ortleppi* develops preferentially in bovine lungs, whereas *E. granulosus* cysts are located in the liver and lungs [8, 24–26]. Therefore, *E. granulosus* and *E. ortleppi* infections in bovines offer the opportunity to analyze two related species with different degrees of adaptation to a single host species.

Molecular characterization of the HF content is essential for a better understanding of *Echinococcus* spp infections. Proteomic studies of helminth ES products have been particularly valuable for identifying proteins involved in the host-parasite relationship [27–29]. Previous proteomic studies of *Echinococcus* ES products included analysis of different *E. granulosus* cyst components [12, 30], comparisons among hydatid cyst fluid of *E. granulosus* cysts from different hosts (sheep, cattle, and humans) [13], and comparison of HF from two different isolates of *Echinococcus multilocularis*, the etiological agent of alveolar echinococcosis [31]. Within the genus *Echinococcus*, proteomic studies involving interspecies comparisons have been performed only between *E. granulosus* and *E. multilocularis* [32]. These studies showed that analyses of the same species infecting different hosts and different genotypes/species/strains infecting a common host can provide valuable insight into molecular survival strategies adopted by parasites. The discovery of proteins shared by distinct species allows identification of conserved mechanisms involved in their interactions with the respective hosts. Furthermore, a species-specific set of proteins can provide molecular markers for parasite diagnosis.

In the present study, we generated MS protein profiles of HF samples from *E. granulosus* and *E. ortleppi* cattle pulmonary cysts. The identified proteins outlined a variety of molecular processes acting in cystic echinococcosis, helping to better understand different aspects of the infection, including parasite survival strategies and host defenses. The generated results will assist the selection of potential targets for new therapeutic approaches and of disease markers capable of differentiating between the two etiological agents.

## Methods

### Biologic material

*Echinococcus ortleppi granulosus* and *E. ortleppi* hydatid cysts were from lungs of cattle obtained at a commercial abattoir in the metropolitan region of Porto Alegre, RS (Brazil). Animal slaughtering was conducted according to Brazilian laws and under the supervision of the *Serviço de Inspeção Federal* (Brazilian Sanitary Authority) of the Brazilian *Ministério da Agricultura, Pecuária e Abastecimento*. Contaminated viscera, identified during mandatory meat inspection, were donated by the abattoir for use in this work.

Lungs were dissected, and HF was aspirated from the hydatid cysts. The HF recovered from individual cysts was centrifuged at 10,000 ×*g* for 15 min at 4 °C to sediment PSCs and debris [12]. Only HF samples from fertile cysts, i.e. with viable PSCs, were used in the study. The PSC DNAs were used for species identification by high-resolution melting (HRM), using a 444-bp fragment of the cytochrome c oxidase subunit I (cox1) gene, and the amplification was carried out with the primers 5′-TTTTTTGGGCATCCTGAGGTTTAT-3′ (forward) and 5′-TAAAGAAAG AACATAATGAAAATG-3′ (reverse), as previously described [33]. Thirty-four *E. granulosus* and 29 *E. ortleppi* HF samples were qualitatively evaluated using 12% SDS-PAGE gel. The intensity of the bovine albumin band, estimated by using IMAGEJ (https://imagej.nih.gov/ij/) to quantify band intensity, was correlated to the cyst volumes. Spearman's rank correlation test was used to estimate the correlation, as previously described [14] (Additional file 1: Figure S1). We selected three individual *E. granulosus* and three individual *E. ortleppi* HF samples (EG1–3 and EO1–3, respectively) with low quantity of albumin for the proteomic analysis.

### Sample preparation and mass spectrometry analysis

Each HF sample protein concentration was determined using Qubit™ (Thermo Fisher Scientific, Inc., Waltham, MA, USA). Proteins were digested in solution using trypsin and fractionated using strong cation exchange (SCX) [14]. To release peptides, 5 mM phosphate buffer (pH 3.0) was added to the SCX columns with a salt gradient, as follows: 75 mM KCl (fraction A), 125 mM KCl (fraction B), 200 mM KCl (fraction C), 300 mM KCl (fraction D), and 400 mM KCl (fraction E). Each fraction was lyophilized and stored at − 80 °C until liquid chromatography-tandem mass spectrometry (LC-MS/MS) analysis.

The five resulting SCX fractions from each one of the six biological samples were analyzed individually, totalizing 30 LC-MS/MS runs. The tryptic peptide mixture corresponding to each SCX fraction was automatically loaded onto a C18 Jupiter pre-column (Phenomenex; bead diameter 10 μm; 100 μm × 50 mm; Phenomenex, Torrance, CA, USA) by an Easy-nLCII nano HPLC system (Thermo Fisher Scientific, Inc., Waltham, MA, USA) coupled to an LTQ-Orbitrap Velos mass spectrometer (Thermo Fisher Scientific). After loading the samples in solvent A (0.1% formic acid), the peptides were subjected to chromatographic separation in reverse-phase using a C18 AQUA column (Phenomenex; beads diameter 5 μm; 75 μm × 100 mm). Both the pre-column and analytical column were packed in house. The peptides were eluted on a gradient of 5%–35% solvent B (0.1% formic acid in acetonitrile) for 60 min; 35%–85% B for 5 min; 85% B for 5 min; 85%–5% B for 2 min; and 5% B in 13 min, under a flow of 200 nl/min. Spray voltage was set at 1.8 kV and 200 °C, and the mass spectrometer was operated in the positive, data-dependent mode, in which one full MS scan was acquired in the m/z range of 300–1800 followed by MS/MS acquisition using collisional induced dissociation (CID) of the ten most intense ions from the MS scan using an isolation window width of 3 m/z. MS spectra were acquired in the Orbitrap analyzer at 30,000 resolution (at 400 m/z). Dynamic exclusion was defined by a list size of 500 and exclusion duration of 90 s at a repetition intervals of 30 s. For the survey (MS) scan, an automatic gain control (AGC) target value of 1,000,000 and maximum injection time of 100 ms were set whereas the target value for the fragment ion (MS/MS) spectra was set to 10,000 and maximum injection time of 100 ms. The lower threshold for targeting precursor ions in the MS scans was 200 counts per scan. The raw files (*.raw) from the MS and MS/MS spectra were converted to the extension *.mgf (mascot generic format) using the MSconvert software (available at http://proteowizard.sourceforge.net).

### Database search and MS data analysis

For protein identification, the generated LC-MS/MS data were used to search local databases containing the known amino acid sequences from the *E. granulosus* genome assembly (PRJEB121), version WBPS11, available at WormBase ParaSite (http://parasite.wormbase.org), and the *Bos taurus* protein sequences obtained from UniProt/Swiss-Prot (Proteome ID: UP000009136).

Mascot Search Engine v. 2.3.02 (Matrix Science, London, UK) was used for peptide and protein identification. The search parameters consisted of carbamidomethylation as a fixed modification, oxidation of methionine as a variable modification, two trypsin missed cleavage, and a tolerance of 10 ppm for precursor and 1 Da for fragment ions. Ion type was set as monoisotopic, and 2 +, 3 +, and 4 + peptide charges were taken into account.

Peptide and protein identification was validated using Scaffold v. 4.8.7 (Proteome Software Inc., Portland, OR, USA). The peptide identification was accepted if it could be established with > 95% probability. Protein identification was accepted if it could be established at > 99% probability and contained two unique identified peptides. The false discovery rate (FDR) was 0.9% and 0.0% for proteins and peptides, respectively. The mass spectrometry data have been deposited to the ProteomeXchange Consortium via the PRIDE [34] partner repository with the dataset identifier PXD019314 and https://doi.org/10.6019/PXD019314.

Some histones (proteins that are highly conserved in eukaryotes) did not fulfill the criteria of at least two unique peptides when the identifications obtained using each database, *E. granulosus*, or *B. taurus* were compared (Additional File 2: Table S1). Because we were unable to definitively determine their organism of origin, histones H4 (EgrG_000323100 and E1BBP7), H2A (EgrG_002051500 and A0A0A0MP90), and H2B (E1BGW2) were removed from further analysis.

Normalized spectral abundance factor (NSAF), acquired using Scaffold, was used to quantify the differences in protein abundance between samples [35]. To determine statistical differences between *E. granulosus* and *E. ortleppi* shared protein NSAF values, we performed a Student’s *t*-test and *P*-value correction using the Benjamini and Hochberg FDR. A heat map analysis was performed using the Matrix2png web interface (https://matrix2png.msl.ubc.ca/) with NSAF values for all identified proteins.

### Prediction of secretion pathways

The identified parasite proteins were searched for the presence of a secretion signal peptide using SignalP 4.1, PrediSi, and SecretomeP 2.0. The presence of an alternative signal for exportation was verified using SecretomeP 2.0. A protein was considered to contain a classical signal peptide when two of the three software programs detected a signal peptide sequence. Proteins that did not meet this criterion, but showed a neural network score (NN score) > 0.6 in SecretomeP, were considered to be alternatively secreted proteins. Those that did not meet any of the previous parameters comprised the group of proteins with an unidentified secretion pattern.

### Functional annotation

Parasitic and bovine proteins were subjected to Gene Ontology (GO) enrichment analysis. The analysis was performed using the total protein repertoire from each species, using the Cytoscape plugin BiNGO [36]. The ontology files were retrieved from GO database, while Wellcome Trust Sanger Institute (UK) kindly provided the files associated with *E. granulosus* protein annotation. Functional enrichment analyses were performed using hypergeometric distribution and *P*-value correction with Benjamini and Hochberg FDR. Values of *P* ≤ 0.05 were considered statistically significant.

The software ESG (extended similarity group) and PFP (protein function prediction), both available at https://kiharalab.org/web/software.php, were used to functionally annotate proteins with an unknown function [37]. The GO terms predicted for a determined protein were considered valid results when they were identified in both ESG and PFP.

The platform REVIGO (http://revigo.irb.hr/) was used to remove redundant GO terms and summarize GO term lists [38]. The semantic similarity of the GO terms was calculated using SimRel (allowed similarity = 0.5).

## Results

### Protein profiles from *E. granulosus* and *E. ortleppi* hydatid fluid samples

**Fig. 1:**
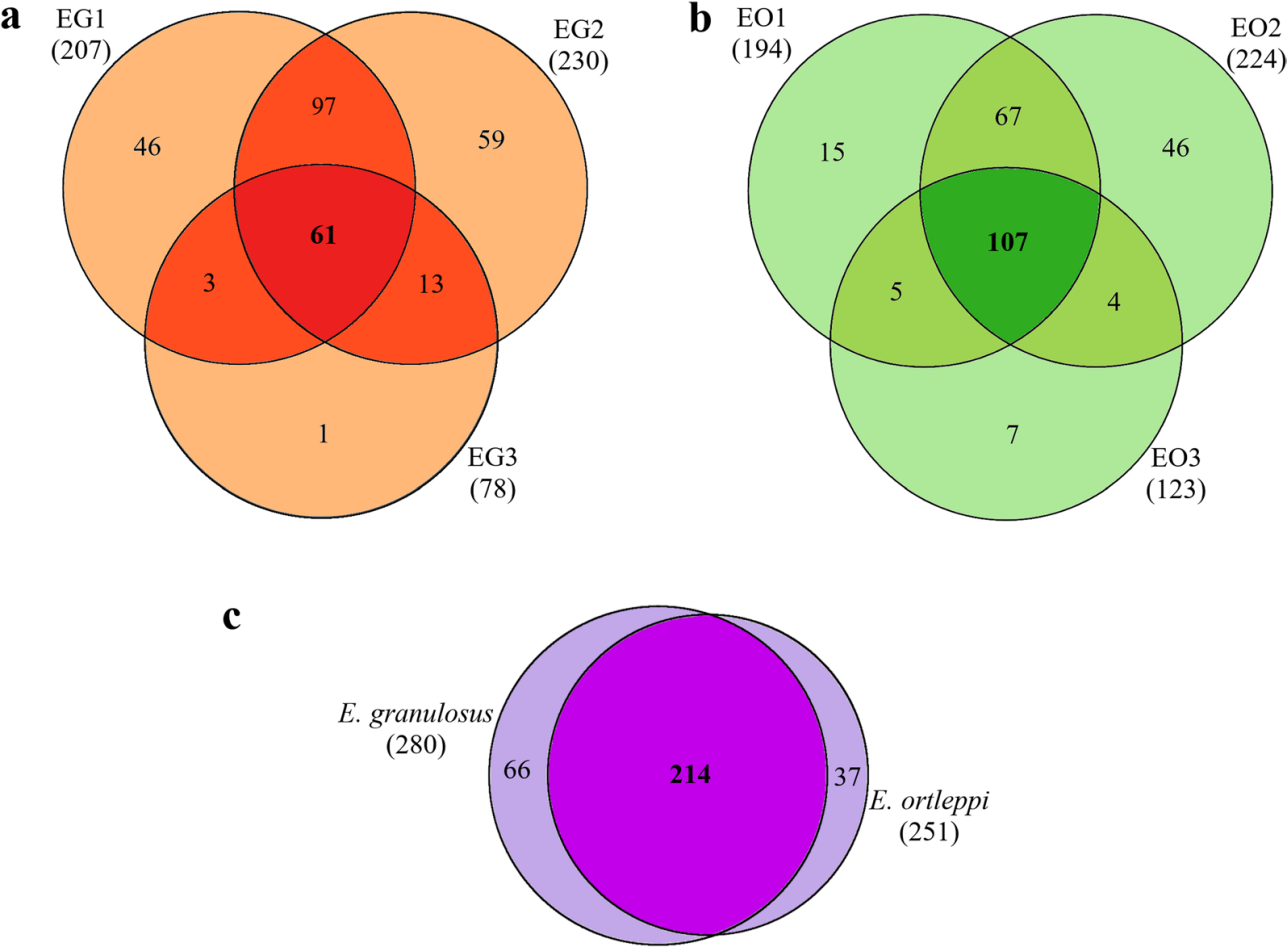
Parasitic proteins identified in HF samples from pulmonary cystic echinococcosis. Venn diagrams showing the number of proteins identified: **a** in *E. granulosus* biological replicates; **b** in *E. ortleppi* biological replicates; **c** in each species or shared between them. The overall numbers of proteins detected are indicated below the sample/species identification

A proteomic survey was performed to describe the HF protein components of *E. granulosus* and *E. ortleppi*. Because *E. ortleppi* develops predominantly in lungs [8, 25] and to minimize differences in the protein profiles due to hydatid cyst location or the host species, we only used samples from pulmonary bovine infections. The number of identified proteins in the three biological replicates, i.e. HF samples from individual fertile hydatid cysts (EG1–3, for *E. granulosus*; EO1–3, for *E. ortleppi*) are summarized in Fig. 1. To visualize the overall sample composition, a heat map analysis was performed using NSAF values of all identified proteins (Additional File 3: Figure S2). The number of proteins identified varied among individual samples from each species. We identified 207, 230, and 78 parasitic proteins in EG1, EG2, and EG3 HF samples, respectively, and overall, 280 *E. granulosus* unique proteins were identified (Additional File 4: Table S2A). In *E. ortleppi* HF samples, we identified 251 unique parasitic proteins, of which 194 were found in EO1, 224 in EO2, and 123 in EO3 (Additional File 4: Table S2B). Overall, 214 proteins were shared between *E. granulosus* and *E. ortleppi*, 66 proteins were found exclusively in *E. granulosus*, and 37 proteins were found exclusively in *E. ortleppi*, totaling 317 proteins. Exclusive proteins identified in *E. granulosus* and *E. ortleppi* are shown in Tables 1 and 2, respectively. Proteins in the shared group did not show differences in abundance between *E. granulosus* and *E. ortleppi*, indicating that the two species may employ similar molecular strategies at the host-parasite interface (Additional File 3: Figure S2 and Additional File 5: Table S3).

**Table 1:** Proteins exclusively identified in HF samples from *E. granulosus* hydatid cysts

Accession number^1^	Protein Name	NSAF^2^	SD^3^	GO terms associated
EgrG_002002600	Alpha mannosidase 2	0.000213	0.000368	Catalytic activity; carbohydrate metabolic process
EgrG_000888900	Anosmin 1	0.000153	0.000266	Regulation of peptidase activity
EgrG_001134100	Aspartate aminotransferase mitochondrial	0.001567	0.002201	Catalytic activity; transferase activity
EgrG_000297300	BC026374 protein S09 family	0.000176	0.000304	Hydrolase activity
EgrG_000741700	Beta-galactosidase	0.000275	0.000476	Carbohydrate metabolic process; hydrolase activity
EgrG_000678900	Bifunctional heparan sulfate	0.000216	0.000374	Hydrolase activity; transferase activity
EgrG_000887000	Cadherin	0.000264	0.000458	Cell adhesion; calcium ion binding
EgrG_000722600	Calcium binding protein	0.000139	0.000241	Calcium ion binding
EgrG_000904400	Carbonic anhydrase	0.000401	0.000694	Carbonate dehydratase activity
EgrG_000477200	Cathepsin L	0.000249	0.000432	Cysteine-type peptidase activity
EgrG_000989200	Cathepsin L1; cathepsin L cysteine peptidase	0.000777	0.001346	Cysteine-type peptidase activity
EgrG_000644850	Cell adhesion molecule	0.000731	0.000634	Protein binding
EgrG_000111700	Complement C1q tumor necrosis factor	0.000801	0.001387	Protein binding
EgrG_000654600	Cysteine protease	0.001284	0.002224	Cysteine-type peptidase activity
EgrG_000061600	Discoidin domain containing receptor 2	0.000232	0.000201	Integral component of membrane
EgrG_001069200	Ectonucleotide pyrophosphatase:phosphodiesterase	0.000200	0.000346	Lipid metabolic process; catalytic activity
EgrG_001096100	EF hand domain containing protein	0.000200	0.000174	Metal ion binding
EgrG_000524400	EGF region	0.000304	0.000527	Cell communication
EgrG_000824100	Estrogen regulated protein EP45; Serpin B9	0.000207	0.000358	Extracellular space
EgrG_000227300	Expressed conserved protein	0.001550	0.002684	–
EgrG_000656900	Expressed conserved protein	0.000446	0.000773	Integral component of membrane
EgrG_000814100	Expressed conserved protein	0.000502	0.000870	Integral component of membrane
EgrG_000956500	Expressed conserved protein	0.000755	0.001307	–
EgrG_000647100	Expressed protein	0.001002	0.001736	–
EgrG_000253000	Glutaminyl peptide cyclotransferase	0.000615	0.000554	Transferase activity
EgrG_000768900	Glycosyl transferase family 8	0.000220	0.000382	Transferase activity
EgrG_000418900	Glycosyltransferase 14 family member	0.000319	0.000553	Transferase activity
EgrG_000778400	Glypican	0.000231	0.000201	Regulation of signal transduction
EgrG_000545700	Hexosyltransferase	0.001173	0.002031	Transferase activity; protein glycosylation
EgrG_000655200	Inositol monophosphatase	0.000277	0.000479	Phosphatidylinositol phosphorylation
EgrG_000357600	Lipase	0.001776	0.001790	Hydrolase activity; lipid catabolic process
EgrG_001157000	Lymphocyte antigen 75	0.000103	0.000178	Integral component of membrane; carbohydrate binding
EgrG_000116900	Lysosomal protein NCU G1 B	0.002407	0.003044	Integral component of membrane
EgrG_000144800	N acyl phosphatidylethanolamine hydrolyzing	0.000477	0.000826	Hydrolase activity
EgrG_000115400	N/A	0.000609	0.001056	Integral component of membrane
EgrG_000237600	N/A	0.000433	0.000750	Integral component of membrane
EgrG_000334500	N/A	0.000566	0.000981	–
EgrG_000759860	N/A	0.000430	0.000744	Integral component of membrane
EgrG_000522900	Neurexin 1 alpha	0.000139	0.000120	Integral component of membrane; multicellular organism development
EgrG_000119200	Neuroendocrine protein 7b2	0.000410	0.000710	Neuropeptide signaling pathway, regulation of proteolysis
EgrG_000926700	Peptide methionine sulfoxide reductase	0.000926	0.001604	Oxidoreductase activity
EgrG_000591200	Pfam-B_8122 and DUF4381 domain containing protein	0.000199	0.000345	Integral component of membrane
EgrG_000443300	Procollagen lysine2 oxoglutarate 5 dioxygenase	0.000119	0.000205	Oxidoreductase activity
EgrG_000443800	Prohormone 4	0.001334	0.001175	Protein binding
EgrG_001022300	Protein disulfide-isomerase	0.000121	0.000210	Isomerase activity
EgrG_000211300	Protein Wnt	0.000167	0.000290	Signaling receptor binding
EgrG_000228100	Protocadherin	0.000141	0.000245	Cell adhesion
EgrG_000861900	Protocadherin 11; Protocadherin-11 X-linked	0.000111	0.000192	Cell adhesion
EgrG_000878500	Protocadherin 9	0.000138	0.000239	Cell adhesion
EgrG_000112900	Protocadherin alpha 6	0.000196	0.000340	Cell adhesion
EgrG_000075800	Receptor protein-tyrosine kinase	0.000082	0.000143	Protein kinase activity
EgrG_000461400	Receptor protein-tyrosine kinase	0.000286	0.000301	Protein kinase activity
EgrG_000655700	Receptor type tyrosine protein phosphatase	0.000131	0.000226	Phosphatase activity
EgrG_000136400	Semaphorin 5B	0.000144	0.000250	Semaphorin receptor binding; multicellular organism development
EgrG_000961100	Slit 2 protein	0.000056	0.000097	Calcium ion binding; multicellular organism development
EgrG_001127800	Speract scavenger receptor	0.000382	0.000661	Scavenger receptor activity; endocytosis
EgrG_000814400	Subfamily M14A unassigned peptidase	0.000222	0.000384	Hydrolase activity
EgrG_000381100	Tapeworm specific antigen B (AgB8/2)	0.026035	0.045093	–
EgrG_000381600	Tapeworm specific antigen B (AgB8/3)	0.017258	0.029891	–
EgrG_000381400	Tapeworm specific antigen B (AgB8/4)	0.009642	0.016701	–
EgrG_000381800	Tapeworm specific antigen B (AgB8/5)	0.002018	0.003496	–
EgrG_000178100	TGF beta family	0.000784	0.000923	Signal transduction
EgrG_000359800	Thioredoxin fold	0.000451	0.000782	–
EgrG_000092800	Transaldolase	0.000264	0.000457	Carbohydrate metabolic process; transferase activity
EgrG_001004900	Transgelin	0.000179	0.000311	Protein binding
EgrG_000959800	Voltage dependent calcium channel subunit	0.000430	0.000572	Calcium channel activity

^a^According to *E. granulosus* genome annotation (PRJEB121, version WBPS11) available at WormBase ParaSite

*NSAF* Normalized spectral abundance factor, *SD* standard deviation

**Table 2:** Proteins exclusively identified in HF samples from *E. ortleppi* hydatid cysts

Accession number^a^	Protein name	NSAF	SD	GO terms associated
EgrG_001104800	Acidic leucine-rich nuclear phosphoprotein	0.000557	0.000964	Protein binding
EgrG_000528900	Actin depolymerizing factor	0.000600	0.001040	Actin cytoskeleton
EgrG_000501600	Alpha-1,4 glucan phosphorylase	0.000193	0.000334	Carbohydrate metabolic process; transferase activity
EgrG_000041200	Annexin	0.000823	0.001426	Calcium ion binding
EgrG_000193700	Annexin	0.001538	0.001436	Calcium ion binding
EgrG_000244000	Annexin	0.001183	0.002048	Calcium ion binding
EgrG_000911200	Calpain-A	0.000366	0.000321	Calcium-dependent cysteine-type endopeptidase activity
EgrG_000936600	Cytoskeleton associated protein CAP Gly containing ankyrin repeats	0.000202	0.000349	Protein binding
EgrG_000564000	Diagnostic antigen gp50	0.000620	0.001074	–
EgrG_000566700	Diagnostic antigen gp50	0.001858	0.001630	–
EgrG_000940900	Dynein light chain	0.001240	0.002149	Microtubule-based process
EgrG_000941100	Dynein light chain	0.005642	0.001419	Microtubule-based process
EgrG_000946900	Dynein light chain	0.000674	0.001168	Microtubule-based process
EgrG_000113800	Elongation factor 1-gamma; eukaryotic translation elongation factor 1	0.000196	0.000339	Translation elongation factor activity
EgrG_000865300	Elongation factor 2	0.000097	0.000168	Translation elongation factor activity
EgrG_000261600	Fructose 1,6 bisphosphatase 1	0.000222	0.000384	Carbohydrate metabolic process; phosphatase activity
EgrG_000476900	GDP L fucose synthase	0.000725	0.000636	Nucleotide-sugar biosynthetic process
EgrG_000882300	Gelsolin; Severin	0.002067	0.001813	Actin filament binding
EgrG_000485800	H17g protein tegumental antigen	0.000937	0.000270	Actin binding; localization of cell
EgrG_002016600	Histone	0.001589	0.001433	DNA binding
EgrG_000906000	Histone H1 delta	0.002786	0.003000	DNA binding
EgrG_000799300	Insulin growth factor binding; Kazal-type serine protease inhibitor domain-containing protein	0.001389	0.002406	Regulation of cell growth
EgrG_000634800	L-lactate dehydrogenase	0.000458	0.000793	Carbohydrate metabolic process; oxidoreductase activity
EgrG_000142500	Major vault protein	0.000456	0.000472	Protein binding
EgrG_000631600	N/A	0.000348	0.000603	–
EgrG_000838600	N/A	0.006931	0.006472	–
EgrG_000736050	NAD(P)H-hydrate epimerase	0.000214	0.000370	Isomerase activity
EgrG_000763300	Paramyosin	0.000714	0.000715	Myosin complex
EgrG_000334550	Peptidylprolyl isomerase	0.001265	0.002192	Isomerase activity
EgrG_000943900	Phosphoglucomutase	0.000901	0.000783	Carbohydrate metabolic process
EgrG_000122100	Profilin	0.002285	0.003957	Actin binding
EgrG_001046200	Subfamily S1A unassigned peptidase S01 family	0.001233	0.001069	Serine-type peptidase activity
EgrG_000607900	Superoxide dismutase	0.000372	0.000645	Superoxide metabolic process
EgrG_001001800	Tegumental antigen	0.000490	0.000848	Microtubule-based process
EgrG_000355700	Tetraspanin	0.000445	0.000771	Integral component of membrane
EgrG_000471600	Transitional endoplasmic reticulum ATPase	0.000103	0.000179	ATPase activity
EgrG_000416400	Triosephosphate isomerase	0.002899	0.000913	Glycolytic process; isomerase activity

^a^According to *E. granulosus* genome annotation (PRJEB121, version WBPS11) available at WormBase ParaSite

*NSAF* Normalized spectral abundance factor, *SD* standard deviation

A large group of proteins of unknown function (35 unique sequences) was identified (Additional File 4: Table S2). They were annotated as “expressed conserved protein,” “expressed protein,” or “N/A (non-annotated).” Some of these proteins of unknown function were identified in all six samples, and some are among the most abundant proteins considering each species separately.

The sequences of proteins of unknown function were subjected to automated function prediction using ESG and PFP software [37]. For ten of these proteins, GO terms predicted by ESG software were further predicted in PFP, and these results are listed in Additional File 6: Table S4. Some molecular function ontologies predicted were calcium channel regulator activity (EgrG_000236300 and EgrG_000296900), RNA binding (EgrG_000316400), DNA binding (EgrG_000471400), and acetylcholine receptor binding (EgrG_000956500). For biological process, chemical synaptic transmission (EgrG_000236300 and EgrG_000296900), regulation of neurotransmitter receptor activity (EgrG_000956500), synapse organization (EgrG_001058700), and protein transport (EgrG_001024500) were some of the ontologies predicted.

As expected, host proteins were also identified in *E. granulosus* and *E. ortleppi* HF samples. Fewer host proteins were identified compared to parasite proteins. Overall, 58 distinct *B. taurus* proteins were identified, with 40 (13 exclusive) of them being identified in *E. granulosus* HF samples and 45 (18 exclusive) in *E. ortleppi* samples, and 27 proteins were common to both samples (Additional File 7: Table S5). Variable numbers of bovine proteins were found in each biological sample, 12, 13, and 28 for EG1, EG2, and EG3, respectively, and 21, 11, and 37 for EO1, EO2, and EO3, respectively (Additional File 8: Figure S3).

### Main proteins identified in hydatid fluid samples from *E. granulosus* and *E. ortleppi*

To highlight the most frequent parasitic proteins in HF, we selected those detected in at least two samples of each species, totaling 217 proteins, among which 13 and 15 were detected exclusively in *E. granulosus* and *E. ortleppi* samples, respectively (Additional File 9: Table S6). For each species, the proteins detected in the three biological samples were selected as HF common proteins (Additional File 10: Table S7). The *E. granulosus* and *E. ortleppi* HF common proteins comprised, respectively, 61 and 105 proteins, and 52 were shared by the two species (Table 3).

**Table 3:** Identification and relative abundance of proteins present in HF samples from *E. granulosus* and *E. ortleppi* bovine pulmonary hydatid cysts

Accession number^a^	Protein name	Molecular Mass^b^	EG		EO	
			NSAF	SD	NSAF	SD
EgrG_000144400	Abnormal EMBroygenesis family member emb 9	168 kDa	0.00382	0.00227	0.00254	0.00064
EgrG_000061200	Actin	42 kDa	**0.01910**	0.01909	**0.01722**	0.00630
EgrG_000156400	Aldo keto reductase family 1 member B4	42 kDa	0.00199	0.00101	0.00277	0.00102
EgrG_000704400	Alpha-mannosidase	118 kDa	0.01298	0.00567	0.01019	0.00120
EgrG_000530400	Amine oxidase	84 kDa	0.00932	0.00324	0.00531	0.00204
EgrG_001032200	Aminotransferase class III; Ornithine aminotransferase	46 kDa	0.01093	0.00898	0.00551	0.00226
EgrG_000184900	Antigen 5	55 kDa	**0.04773**	0.00409	**0.05786**	0.02534
EgrG_000575900	Basement membrane specific heparan sulfate	860 kDa	0.01233	0.00292	0.00869	0.00066
EgrG_000701800	Basement membrane specific heparan sulfate	96 kDa	0.00857	0.00187	0.00467	0.00036
EgrG_000879900	Beta D xylosidase 2	92 kDa	0.00252	0.00108	0.00271	0.00146
EgrG_000789900	Beta mannosidase	108 kDa	0.00220	0.00084	0.00115	0.00032
EgrG_000903100	Calsyntenin 1	130 kDa	0.00405	0.00445	0.00285	0.00157
EgrG_000970500	Cathepsin D lysosomal aspartyl protease	47 kDa	**0.01626**	0.00742	**0.01783**	0.00509
EgrG_000144350	Collagen alpha 1(IV) chain	172 kDa	0.00553	0.00266	0.00351	0.00066
EgrG_000417600	Collagen alpha 1(IV) chain	182 kDa	0.00280	0.00195	0.00154	0.00066
EgrG_000203400	Collagen alpha 1(V) chain	172 kDa	0.00630	0.00263	0.00512	0.00185
EgrG_000144300	Collagen alpha 1(V) chain	177 kDa	0.00453	0.00246	0.00309	0.00012
EgrG_000729300	Collagen alpha 1(XV) chain	191 kDa	0.00398	0.00232	0.00250	0.00054
EgrG_000823800	Collagen alpha 2(I) chain	131 kDa	0.00914	0.00326	0.00605	0.00239
EgrG_001190600	Collagen type I II III V XI alpha	123 kDa	0.00786	0.00374	0.00445	0.00175
EgrG_000524200	Collagen type XI alpha 2	163 kDa	0.00445	0.00130	0.00191	0.00115
EgrG_000766600	Cysteine-rich secretory protein LCCL domain-containing; Peptidase inhibitor 16	29 kDa	**0.01633**	0.01258	0.00751	0.00292
EgrG_000255800	EGF domain protein	267 kDa	0.00385	0.00187	0.00193	0.00014
EgrG_000682900	Epididymal secretory protein E1; Niemann Pick C2 protein	20 kDa	0.00778	0.00123	0.00369	0.00151
EgrG_000824000	Estrogen regulated protein EP45	45 kDa	0.01048	0.00136	0.00722	0.00093
EgrG_001061900	Expressed conserved protein	74 kDa	0.00762	0.00602	**0.01483**	0.00569
EgrG_000412500	Expressed conserved protein	14 kDa	**0.01324**	0.00948	**0.01267**	0.00320
EgrG_000523100	Expressed conserved protein	53 kDa	0.00638	0.00255	0.00600	0.00175
EgrG_000596300	Expressed conserved protein	25 kDa	**0.01830**	0.01295	**0.01610**	0.00609
EgrG_000316400	Expressed protein	35 kDa	0.00261	0.00088	0.00217	0.00114
EgrG_000842900	Fgfr protein	80 kDa	0.00302	0.00128	0.00273	0.00042
EgrG_001060700	Fibrillar collagen chain FAp1 alpha	116 kDa	0.00989	0.00164	0.00725	0.00357
EgrG_000176400	Fras1 related extracellular matrix protein	263 kDa	0.00187	0.00107	0.00147	0.00037
EgrG_000905600	Fructose-bisphosphate aldolase	40 kDa	**0.01739**	0.00955	0.01265	0.00119
EgrG_000712600	Gynecophoral canal protein	97 kDa	0.00883	0.00348	0.00577	0.00146
EgrG_000824400	Gynecophoral canal protein; Transforming growth factor-beta-induced protein ig-h3	73 kDa	0.01180	0.00181	0.00923	0.00139
EgrG_000422350	Hemicentin 1	477 kDa	0.00279	0.00100	0.00138	0.00065
EgrG_001132400	Laminin	395 kDa	0.00113	0.00081	0.00054	0.00029
EgrG_000458400	Laminin subunit gamma	163 kDa	0.00254	0.00085	0.00105	0.00044
EgrG_000684200	Lipid transport protein N terminal	344 kDa	0.00439	0.00189	0.00236	0.00186
EgrG_000343000	Neurogenic locus notch protein	339 kDa	0.00555	0.00140	0.00384	0.00030
EgrG_001181950	Papilin	67 kDa	0.00315	0.00094	0.00159	0.00057
EgrG_000920600	Peptidyl-prolyl cis–trans isomerase	17 kDa	0.01078	0.00123	**0.01451**	0.00122
EgrG_000292700	Phosphoenolpyruvate carboxykinase	71 kDa	0.01145	0.00131	**0.01344**	0.00424
EgrG_001132700	Poly(U) specific endoribonuclease	29 kDa	0.00748	0.00254	0.00659	0.00084
EgrG_000849600	Proteinase inhibitor I25 cystatin	31 kDa	**0.03216**	0.01174	**0.02514**	0.01025
EgrG_001133400	Protein-L-isoaspartate O-methyltransferase	27 kDa	0.00630	0.00374	0.00363	0.00209
EgrG_000929500	SPONdin extracellular matrix glycoprotein	111 kDa	0.00293	0.00178	0.00126	0.00016
EgrG_000381200	Tapeworm specific antigen B (AgB8/1)	10 kDa	**0.09772**	0.00805	**0.19553**	0.08363
EgrG_000791700	Thioredoxin peroxidase	21 kDa	**0.01305**	0.00927	0.01254	0.00268
EgrG_001060600	Type II collagen B	154 kDa	0.00352	0.00080	0.00206	0.00116
EgrG_000317300	Vesicular amine transporter	49 kDa	0.01186	0.00156	0.00688	0.00342

The listed proteins were identified in the three biological replicates from each species. Quantitative data are presented based on averaged NSAF values calculated for *E. granulosus* (EG) and *E. ortleppi* (EO)

*NSAF* Normalized spectral abundance factor, *SD* standard deviation

^a^According to *E. granulosus* genome annotation (PRJEB121, version WBPS11) available at WormBase ParaSite

^b^Molecular mass calculated from primary sequence

Top ten NSAF values in HF samples of each species are highlighted in bold

Within the HF common proteins, in the subgroup of proteins shared between the two species, we identified proteins associated with different biological processes, such as cathepsin D, laminin, thioredoxin peroxidase, poly(U) endoribonuclease, cystatin, fructose-bisphosphate aldolase, and antigens previously described as relevant in *Echinococcus* spp. biology, such as antigen B (AgB) and antigen 5 (Ag5). AgB and Ag5 are antigens with recognized significance in *Echinococcus* spp. biology by their abundance and immunogenicity.

AgB is an oligomeric lipoprotein, which can comprise up to five related subunits (AgB8/1 to 5). We detected subunit AgB8/1 in the shared subgroup of common proteins, while subunits AgB8/2 to 5 were detected in only one *E. granulosus* sample (Additional File 3: Figure S2 and Additional File 5: Table S3). These subunit levels in the other samples might be below the level of detection under our experimental conditions.

HF common proteins shared between *E. granulosus* and *E. ortleppi* are interesting study targets to understand molecular mechanisms at the host-parasite interface in cystic echinococcosis. Additionally, they are candidate targets for the development of new therapies for *Echinococcus* spp. infections.

Some *B. taurus* proteins were more frequently identified in our HF analysis. The host proteins found in at least two biological replicates in each species are listed in Table 4. The proteins actin, apolipoprotein A-1, heat shock cognate 71 kDa protein, hemoglobin subunit alpha, hemoglobin subunit beta, and serum albumin were identified in HF samples from both species.

**Table 4:** Bovine proteins identified in at least two biological replicates from *E. granulosus* and *E. ortleppi* hydatid fluid

Accession number^a^	Protein name	Molecular mass^b^	NSAF	SD	GO terms associated
*E. granulosus*					
1433G_BOVIN	14–3-3 protein gamma	28 kDa	0.0148	0.01632	Regulation of biological quality; protein binding
ACTB_BOVIN	Actin, cytoplasmic 1	42 kDa	0.06094	0.01676	Protein binding; response to toxic substance
FETUA_BOVIN	Alpha-2-HS-glycoprotein	38 kDa	0.015	0.01725	Endopeptidase regulator activity; defense response
APOA1_BOVIN	Apolipoprotein A-I	30 kDa	0.0141	0.01228	Protein binding; regulation of protein transport
HSP7C_BOVIN	Heat shock cognate 71 kDa protein	71 kDa	0.0036	0.0032	Nucleotide metabolic process; protein binding
HBA_BOVIN	Hemoglobin subunit alpha	15 kDa	0.19286	0.05119	Detoxification; cellular response to chemical stimulus
HBB_BOVIN	Hemoglobin subunit beta	16 kDa	0.27648	0.05495	Detoxification; cellular response to chemical stimulus
A0A140T897_BOVIN	Serum albumin	69 kDa	0.19232	0.10116	Protein binding; cell killing
*E. ortleppi*					
ACTB_BOVIN	Actin, cytoplasmic 1	42 kDa	0.09472	0.05639	See above
ENOA_BOVIN	Alpha-enolase	47 kDa	0.0036	0.00314	Glycolytic process; binding
APOA1_BOVIN	Apolipoprotein A-I	30 kDa	0.011	0.00995	See above
CATA_BOVIN	Catalase	60 kDa	0.0048	0.00521	Cellular response to toxic substance; detoxification
HSP7C_BOVIN	Heat shock cognate 71 kDa protein	71 kDa	0.00934	0.00643	See above
HBA_BOVIN	Hemoglobin subunit alpha	15 kDa	0.20497	0.01557	See above
HBB_BOVIN	Hemoglobin subunit beta	16 kDa	0.33547	0.06589	See above
LDHA_BOVIN	L-lactate dehydrogenase A chain	37 kDa	0.0107	0.00933	Carbohydrate metabolic process
F1MYX5_BOVIN	Lymphocyte cytosolic protein 1	70 kDa	0.0028	0.00247	Immune response; regulation of localization
PRDX1_BOVIN	Peroxiredoxin-1	22 kDa	0.0125	0.01226	Immune response; detoxification
A5D984_BOVIN	Pyruvate kinase	58 kDa	0.0034	0.00292	Glycolytic process; binding
A0A140T897_BOVIN	Serum albumin	69 kDa	0.09619	0.02085	See above
TBA1B_BOVIN	Tubulin alpha-1B chain	50 kDa	0.0049	0.00441	Nucleotide binding
TBB5_BOVIN	Tubulin beta-5 chain	50 kDa	0.0051	0.00475	Nucleotide binding
VIME_BOVIN	Vimentin	54 kDa	0.0128	0.01761	Immune response; cellular response to chemical stimulus

^a^According to *Bos taurus* reference proteome (ID: UP000009136) available at Uniprot/Swiss-Prot

^b^Molecular mass calculated from primary sequence

*NSAF* Normalized spectral abundance factor, *SD* standard deviation

### Potential secretion pathways associated with parasitic proteins identified in *E. granulosus* and *E. ortleppi* hydatid fluid

**Fig. 2:**
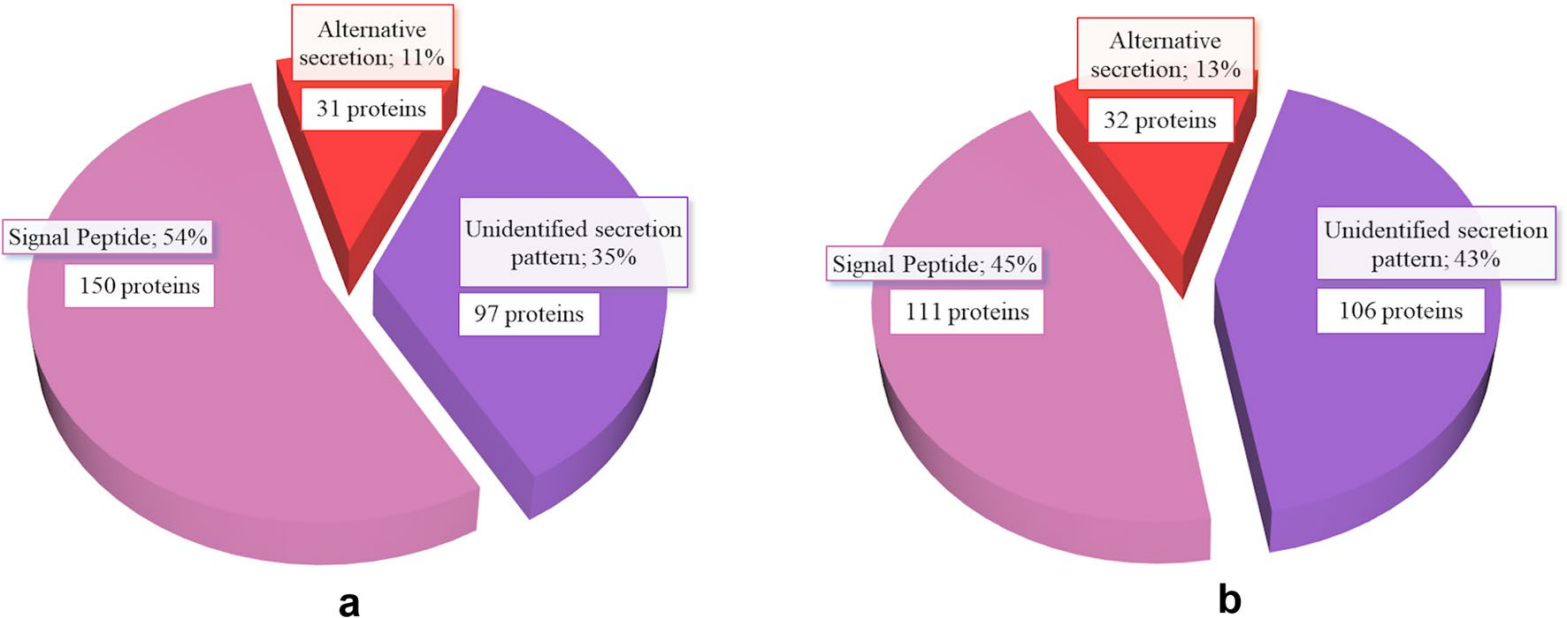
In silico prediction of secretion pathways. Percentages of the total and absolute number of proteins in *E. granulosus* and *E. ortleppi* HF repertoires with probable classic or alternative signals for secretion are presented. Proteins with any identifiable signal for secretion were grouped under the term “Unidentified secretion pattern.” **a** *E. granulosus* HF proteins. **b** *E. ortleppi* HF proteins

All *E. granulosus* and *E. ortleppi* proteins identified in the corresponding HF samples were analyzed using bioinformatic tools to predict whether they would be secreted by a classical pathway (signal peptide) or by an alternative pathway, and the results are summarized in Fig. 2. In the *E. granulosus* protein repertoire (Fig. 2a), 54% (150/278) of the proteins were predicted to have a signal peptide, 11% (31/278) were predicted to be secreted by an alternative pathway, and 35% (97/278) were not predicted to be secreted. In the *E. ortleppi* repertoire (Fig. 2b), 45% (111/249) of the proteins were predicted to have a signal peptide, 13% (32/249) were predicted to be secreted by an alternative pathway, and 43% (106/249) were not predicted to be secreted.

### Functional annotation of the protein repertoires from *E. granulosus* and *E. ortleppi* hydatid fluid

GO enrichment analyses were performed for all parasitic proteins identified in *E. granulosus* and *E. ortleppi* using the Cytoscape plugin BiNGO [36]. Functional classification with GO enrichment data is shown in Additional File 11: Table S8. Most proteins were functionally annotated for both *E. granulosus* (220/278 proteins) and *E. ortleppi* (203/249 proteins). GO enrichment (*P* ≤ 0.05) was found for 180 GO subcategories in *E. granulosus* (Additional File 11: Table S8A) and for 224 GO subcategories in *E. ortleppi* (Additional File 11: Table S8B), using the following three main GO categories: biological process, molecular function, and cellular component. *Echinococcus granulosus* and *E. ortleppi* showed the same profile regarding the most significant GO subcategories (*P* < 0.001).

**Fig. 3:**
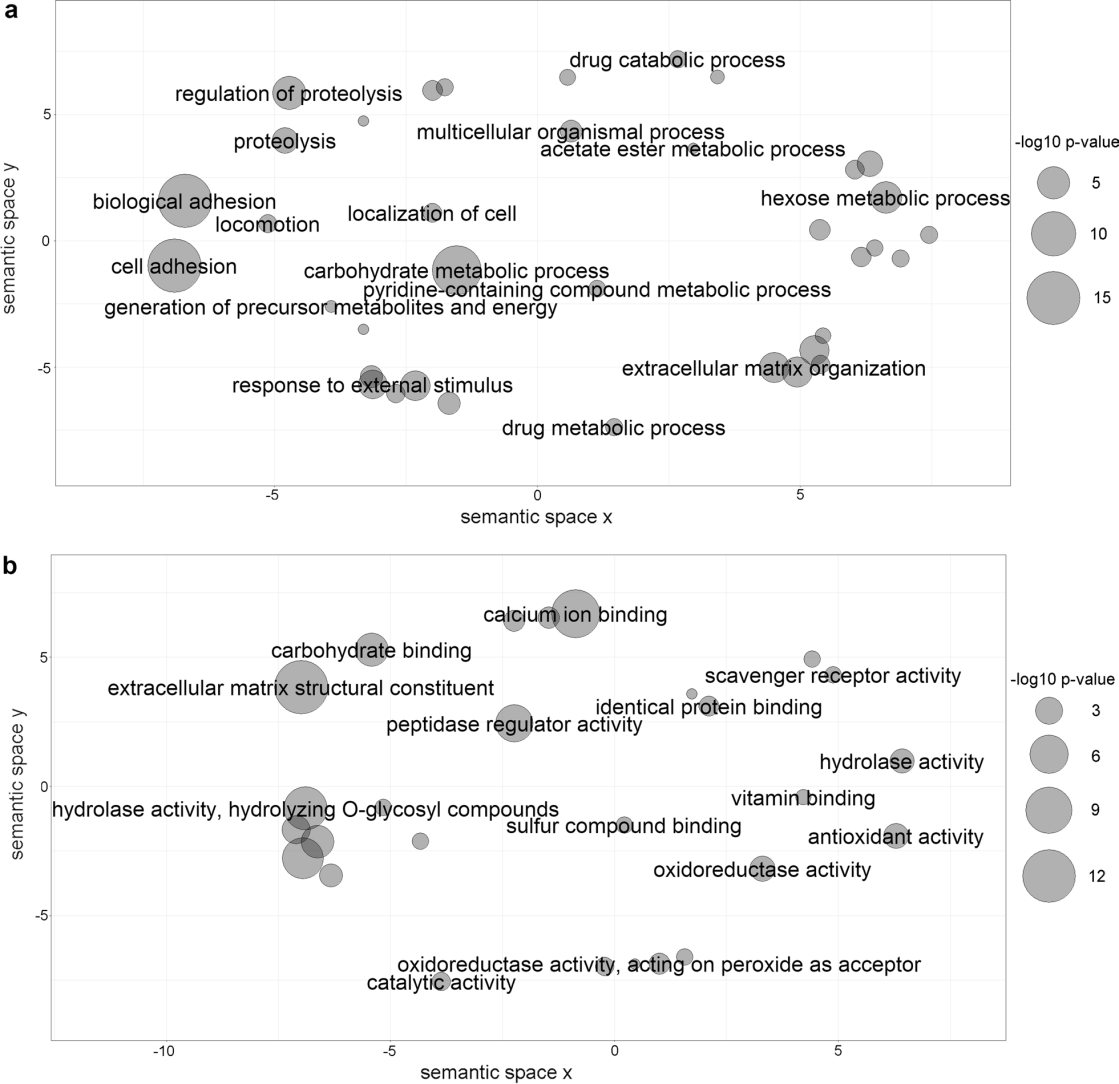
Summarized functional classification of proteins identified in *E. granulosus* HF. Scatterplot view of REVIGO category clusters of related GO terms obtained in functional enrichment analysis. **a** Biological process category clusters. **b** Molecular function category clusters. Sphere size is proportional to the *P*-value (larger spheres indicate more significant *P*-values, according to the scale)

**Fig. 4:**
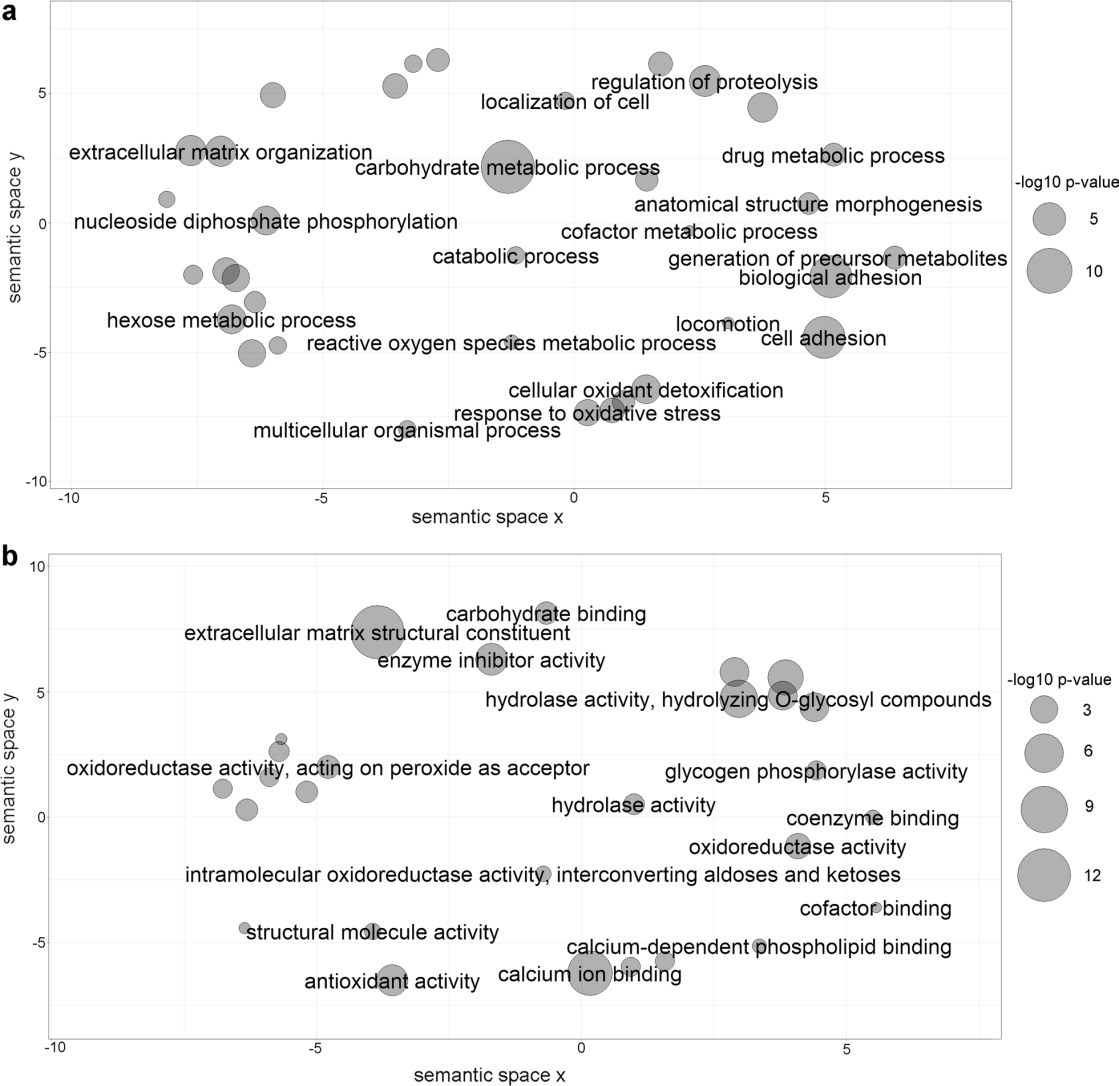
Summarized functional classification of proteins identified in *E. ortleppi* HF. Scatterplot view of REVIGO category clusters of related GO terms obtained in functional enrichment analysis. **a** Biological process category clusters. **b** Molecular function category clusters. Sphere size is proportional to the *P*-value (larger spheres indicate more significant *P*-values, according to the scale)

The enriched GO terms in the biological process and molecular function major categories for *E. granulosus* and *E. ortleppi* proteins were summarized using REVIGO [38]. The complete lists of summarized non-redundant terms are shown in Additional File 12: Table S9 and Additional File 13: Table S10. After the summary using REVIGO, 65 and 63 category clusters were generated for *E. granulosus* and *E. ortleppi*, respectively. For the biological process main category, the clusters “cell adhesion,” “carbohydrate metabolic process,” and “regulation of proteolysis” were among the most enriched clusters in both *E. granulosus* and *E. ortleppi* (Figs. 3a and 4a). In the molecular function main category, the clusters “extracellular matrix structural constituent,” “calcium binding,” and “hydrolase activity acting on glycosyl bonds” were among the most enriched clusters in both *E. granulosus* and *E. ortleppi* (Figs. 3b and 4b).

Host proteins identified in *E. granulosus* and *E. ortleppi* HF samples were also subjected to GO enrichment analysis. An extensive list of GO terms were enriched (281 in *E. granulosus* and 378 in *E. ortleppi*), and they were summarized using the REVIGO platform (Additional File 14: Table S11 and Additional File 15: Table S12).

REVIGO category clusters generated for the bovine proteins in *E. granulosus* and *E. ortleppi* HF showed different host biological mechanisms. There were “carbohydrate metabolic process,” “defense response,” “cell killing,” “protein binding,” and “regulation of protein stability” among the shared category clusters. Some *E. granulosus* category clusters were “negative regulation of hydrolase activity,” “acute-phase response,” and “regulation of peptide transport.” Some *E. ortleppi* category clusters were “response to external stimulus,” “immune response,” and “regulation of cell death.”

## Discussion

In our study, we performed a MS-based proteomic analysis of HF samples from three *E. granulosus* and three *E. ortleppi* hydatid cysts collected from *B. taurus* lungs. We identified 280 and 251 proteins in *E. granulosus* and *E. ortleppi* samples, respectively, and there were 317 different parasitic proteins overall.

Many proteins identified in our study do not have a signal to secretion, and because of that, they would be unexpected in HF. However, extracellular vesicles are described in the literature as carriers for a wide range of proteins, indicating that proteins without recognizable signal peptide can also be secreted to exert their function extracellularly. The composition of the extracellular vesicles is diverse, including several classes of proteins, like signaling proteins, membrane receptors, glycolytic enzymes, proteases, inhibitors, etc. A quick search in exocarta (http://exocarta.org) and vesiclepedia (http://www.microvesicles.org/) databases showed that several proteins from the HF repertoire of *E. granulosus* and *E. ortleppi* have been identified in extracellular vesicles from other organisms. Proteomic analyses of *E. granulosus* extracellular vesicles isolated from sheep [39] and human hosts [40] have shown several proteins in common with our results, supporting that this may be the mechanism of secretion for many proteins in *Echinococcus* metacestode.

The GO analysis showed the association of the protein profiles with a variety of ontology terms. The heterogeneity of functions assigned to the identified proteins, such as cell adhesion, extracellular matrix structural constituent, carbohydrate metabolic process, and calcium binding, indicates that many molecular mechanisms are active in *E. granulosus* and *E. ortleppi* larval infection. The heterogeneity in the function and number of proteins in our samples may result from differences in the cyst developmental stage or their physiological state.

Proteins associated with nutrient transport and metabolism were well represented in our analysis. These proteins may act in basic cellular functions, playing important roles in nutrient uptake and in structural constituent and energy production. Some of them were found in both *E. granulosus* and *E. ortleppi* HF protein repertoires, such as beta mannosidase, fructose-bisphosphate aldolase, phosphoenolpyruvate carboxykinase, aminotransferase class III, AgB, and lipid transport protein N terminal. The metacestode is very active and certainly requires a good supply of nutrients and energy to maintain the viability. Tapeworms have reduced synthesis capability, but an increased ability to absorb nutrients from host [41, 42]. *Echinococcus* do not synthesize fatty acids and cholesterol; instead, they scavenge them from the host. AgB is a lipoprotein acting in transport of host-derived fatty acids, triacylglycerols, and sterols to the parasite tissues [43, 44]. AgB is also the major antigen in HF, and it has important immunomodulatory properties [11]. Among all five AgB subunits, we detected only AgB8/1 in all six analyzed samples. AgB8/1 has been reported to be the most abundant subunit in the *E. granulosus* AgB oligomer [45], and the AgB subunit is consistently identified by MS-based HF analysis [12, 14, 32]. However, detection and abundance of AgB8/2–5 in HF are variable [12–14, 32]. A few works have analyzed the proportion of each subunit in the AgB pool in HF, and many questions remain unanswered, such as the dynamic of subunit production along the metacestode development or whether the production is modulated upon determined host responses. Additionally, AgB subunit representation in HF varies among different *Echinococcus* species and isolates in *E. multilocularis*, for example AgB8/3 is the most abundant subunit [32, 46].

Different carbohydrate-metabolizing enzymes were identified in *E. granulosus* and *E. ortleppi* HF. Such enzymes are repeatedly observed in the secretome of *E. granulosus* and other cestodes, including *E. multilocularis* [14, 31, 47]. In *E. granulosus*, they have been found in the HF of hydatids from cattle, sheep and human hosts [13, 14, 32, 48]. Previous studies indicated that some carbohydrate-metabolizing enzymes exerted other effects in addition to their primary biochemical roles [49]. In addition to their described function, the carbohydrate-metabolizing enzymes identified in this study might exert extracellular functions, protecting parasite tissues from host immune attack and aiding in metacestode development. Glycolytic enzymes were shown to exert many effects, such as binding to complement proteins and interference in their response, binding of host plasminogen with further increase in its activation and interaction with adhesins and the cytoskeleton to facilitate invasion [50–52]. In *E. granulosus*, fructose-bisphosphate aldolase was shown to interact with actin, and enolase was detected by immunolocalization in the laminated layer of hydatids from cattle [17]. These molecules do not have a signal peptide, but significant amounts appeared to be secreted through specific mechanisms such as extracellular vesicles [14, 17]. The glycolytic enzymes have been identified in extracellular vesicles of HF from sheep and human hydatids [39, 40].

*Echinococcus ortleppi granulosus* and *E. ortleppi* HF showed a diverse range of proteolytic enzymes. Our analysis identified enzymes such as aminopeptidases, carboxypeptidases, cysteine peptidases, metalloproteases, and an enteropeptidase. Proteolytic enzymes have pivotal roles at the host-parasite interface, especially related to nutrient acquisition, tissue migration, and protection against the host immune response [53–56]. Metalloproteases, a class of proteolytic enzymes frequently found in parasitic secretomes, function mainly in extracellular matrix degradation and tissue remodeling, and they also facilitate a diverse range of cellular processes, including regulation of stem cell proliferation in planarians [57]. Cathepsins are cysteine proteases that are widely described as molecular players in helminthic infections and suppress the host immune response at the host-parasite interface [56]. Three cathepsin L sequences were identified in *E. granulosus*. Calpain, a Ca^2+^-dependent cysteine protease, was identified in *E. ortleppi* HF. Calpains are associated with cell degeneration; studies have reported that under Ca^2+^ imbalances, calpains become activated and mediate apoptosis and necrosis [58–60]. Thus, a role for Calpain-A as a defense molecule inducing cell death at infiltrating and adjacent host cells in *E. ortleppi* infection is possible. Based on their importance in different processes of basic parasitic biology and their role at the host-parasite interface, some proteases have been proposed as therapeutic targets [61–64].

However, protease inhibitors such as cystatins, serpins, and proteins containing Kunitz and Kazal domains were also detected in *E. granulosus* and *E. ortleppi* HF. Proteases are part of defense mechanisms in mammals, and the presence of parasitic protease inhibitors suggests that modulation of host protease activities could be a mechanism of protection against elimination in *Echinococcus* spp. Proteases and inhibitors could also be associated with the same molecular processes in which the inhibitors regulate protease activity to avoid excessive tissue damage [65]. Thus, the parasite would produce inhibitors to modulate their own protease activity to minimize host tissue damage and avoid an increased immune response at the infection site. Important immunomodulatory roles have been described for protease inhibitors in other parasitic flatworms [65, 66]. In different invertebrates, Kunitz proteins have been described as acting in defense against microbial infection and with toxin activity mediated by ion channel blockade [67, 68].

A group of proteins related to the extracellular matrix and structure maintenance was identified in both *E. granulosus* and *E. ortleppi* HF. We highlight the presence of proteins associated with extracellular matrix structures and dynamics, such as collagen, laminin, hemicentin-1, SPONdin extracellular matrix glycoprotein, basement membrane specific heparan sulfate, and FRAS1-related extracellular matrix protein 1. These proteins may be related to maintenance of the hydatid cyst wall structural integrity, helping the metacestode to resist the host responses. The germinative layer inside the hydatid cyst plays a pivotal role in hydatid cyst development and survival, and its outward face is covered by a syncytial tegument that is also a physical barrier against the entrance of macromolecules into hydatid cysts [4, 69]. The laminated layer, an acellular, carbohydrate-rich sheath secreted by the germinative layer, shields the parasite from direct attack by host immune cells [70]. The extracellular matrix proteins and their regulators may be associated with a molecular network that both maintains the integrity of the cyst wall and allows tissue expansion that is necessary for hydatid growth.

Signaling pathway proteins were also identified, and many of them were shared between *E. granulosus* and *E. ortleppi*. Desert hedgehog protein (Dhh), noggin, notch, tyrosine protein kinase otk, and glypican-1 are examples of signaling proteins that play crucial roles in embryonic and morphological development in model organisms such as *Caenorhabditis elegans*, *Drosophila melanogaster*, and *Mus musculus* [71–74]. The germinative layer in fertile metacestodes comprises cells that actively participate in cyst development. These cells differentiate to generate brood capsules and PSC, secrete some HF components, and produce the required molecules to maintain cyst wall integrity [4, 75]. In this work, only viable fertile hydatid cysts were used, and thus, the germinative layer was probably very active and the signaling proteins we found could have a function in coordinating the events in this cell layer.

Some proteins identified here are linked to the major developmental pathways, Hedgehog, Notch, and Wnt, which are involved in many embryological development cascades, cell fate, cell polarity, and maintaining stemness of stem cells [73, 74, 76]. Because some cells in the germinative layer are stem cells responsible for generating other cell types and tissues in the metacestode, our findings suggest that such developmental pathways are active in the *Echinococcus* spp. hydatid cyst. Differential expression of signaling proteins among different *E. granulosus* and *Hymenolepis microstoma* developmental stages has been previously demonstrated [77, 78]. In *E. multilocularis* and *H. microstoma*, Wnt protein expression patterns during larval metamorphosis have been elucidated [79]. The roles played by the signaling transducing proteins might be necessary for proper metacestode development and growth.

Identification of extracellular matrix-related and signaling transduction proteins in the HF compartment indicates that they are secreted by germinative cells, brood capsules, or protoscoleces. Some of these proteins were identified in *E. granulosus* extracellular vesicles isolated from HF of sheep and human hydatid cysts [39, 40]. We hypothesized that production of extracellular vesicles containing these proteins could be a strategy to spread them to the entire cyst wall extension, as a form of coordinating processes at distinct positions in the germinative layer. Germinative layer secretion activity occurs in inward and outward directions in the hydatid cyst, so it is possible that these proteins could also act upon nearby host tissue.

Proteins discussed so far have also been identified by proteomic studies of *E. granulosus* total HF or extracellular vesicles in sheep or human infections [13, 32, 39, 40, 48]. Considering they are produced by *E. granulosus* in different hosts and by *E. ortleppi* too, these classes of proteins, i.e. carbohydrate-metabolizing enzymes, transporters, extracellular matrix-related proteins, signaling proteins, proteases, and inhibitors, seem to have pivotal roles in parasite biology.

Some proteins were identified for the first time, to our knowledge, in the HF from *E. granulosus*, such as speract scavenger receptor, hexosyltransferase, peptide-methionine sulfoxide reductase, ectonucleotide pyrophosphatase:phosphodiesterase, Cupin 2 barrel domain containing protein, armet protein, semaphorin 5B, EF hand domain containing protein, TGF beta family, and structural maintenance of chromosomes protein. These proteins are not characterized in *Echinococcus* sp., but they may represent molecular events associated to the lung location of the metacestode. Peptide-methionine sulfoxide reductase acts in an oxidation-reduction process that might protect the parasite tissues from oxidative damage. Scavenger receptors bind different molecules and facilitate endocytosis in mammals [80]. Semaphorins are involved in vesicular transport in *C. elegans*, which is an important mechanism for cell shape regulation during development [81]. TGF-β/Smad system is described playing a role in parasite tolerance and in liver fibrosis in *E. multilocularis* infection [82], so we reasoned whether the production of TGF beta family proteins could be a mechanism to modulate the fibrotic response in the host organ. These are possibilities that need to be verified in cystic echinococcosis.

We report for the first time a proteomic survey in *E. ortleppi*, the species best adapted to cattle as intermediate host. The exclusive repertoire of proteins identified in *E. ortleppi* HF shows three annexin sequences. There are some indications from studies in other helminths that annexins may act as defense molecules by inducing apoptosis in host immune cells [83–85]. Calpain-A (discussed before) is another protein exclusively found in *E. ortleppi* HF that mediates apoptosis [59, 60]. Higher levels of apoptotic proteins could be a characteristic of *E. ortleppi* to deal with host defenses, resulting in better development in bovine hosts. Further investigations will be necessary to determine the existence of differential patterns of apoptosis between *E. granulosus* and *E. ortleppi*.

The exclusive *E. ortleppi* repertoire has different proteins associated to cytoskeleton dynamics, for example: actin depolymerizing factor, cytoskeleton associated protein CAP, gelsolin, and profilin. These findings are interesting because the HF is an extracellular compartment; the roles of these proteins in the HF could be other than those related to actin and microtubule organization. This possibility needs to be investigated in the future, because currently there is no evidence of the function of these proteins in the HF.

Host proteins were also identified in the HF samples, but there were fewer than in the parasitic proteins. They were diverse among the biological replicates from *E. granulosus* and *E. ortleppi*, with only four bovine proteins identified from all the HF samples. Different classes of host proteins permeate into the hydatid cyst, and, as we highlight for the parasite protein profile, the cyst physiological state or developmental stage may be related to this heterogeneity. Host proteins can be part of the defense mechanisms that act to eliminate the parasite, as indicated by the enriched GO terms “defense response” and “immune response.” However, the parasite may also take up these proteins for its own use. The specific roles of host proteins in fertile HF are currently unknown, and further thorough studies are necessary to unveil them.

The balance of parasite-host protein content in HF has been associated with *E. granulosus* hydatid cyst fertility conditions, where fertile cysts have a predominant protein content from the parasite, while infertile hydatid cysts have a higher protein content from the host [14]. Samples collected in this study were from fertile hydatid cysts, so the low number of host proteins identified is consistent with other studies. Infertile hydatid cysts may have a weakened wall and are more susceptible to host protein entry. We identified a large set of parasitic proteins that are related to extracellular matrix and structure maintenance, which supports the idea that in fertile hydatid cysts, the wall is an important barrier to protect the parasite.

## Conclusions

Our proteomic analysis highlighted proteins involved in molecular mechanisms, such as adhesion, extracellular structures organization, development regulation, signaling transduction, and enzyme activity, which are present at the host-parasite interface during *E. granulosus* and *E. ortleppi* infections in lungs from bovine hosts. The results provide valuable information on the *E. granulosus* and *E. ortleppi* molecular mechanisms during host chronic infection, helping to understand biological aspects of cystic echinococcosis caused by different parasite species. The data contribute to knowledge about *E. ortleppi*, a species that is still poorly characterized molecularly. The observed *E. granulosus* and *E. ortleppi* protein profiles can guide the choice of specific molecular processes to use in further studies on these two species. Some of the identified proteins and the pathways they belong to may be of clinical interest because they can be further explored to develop novel and more effective therapies against these and other *Echinococcus* species.

## Supplementary Information

**Additional file 1: Figure S1.** *E. granulosus* and *E. ortleppi* HF protein comparison. (A) Correlation between cysts volume and intensity of bovine albumin band. Thirty-four *E. granulosus* and 29 *E. ortleppi* HF samples were qualitatively evaluated using 12% SDS-PAGE gel. The intensity of the bovine albumin band, estimated by using IMAGEJ (https://imagej.nih.gov/ij/) to quantify band intensity, was correlated to the cyst volumes. The six HF samples from cysts with similar sizes (4–6 cm diameter) used in the proteomic analysis are indicated by blue squares (*E. granulosus*) and red squares (*E. ortleppi*). (B) Analysis of HF proteins from the selected samples. 50 μg of HF proteins *E. granulosus* (EG1–3) and *E. ortleppi* (EO1–3) samples were evaluated by 12% SDS-PAGE gel. For each sample it was possible to identify stained proteins from 10 to 250 kDa. Markers are indicated on the left.

**Additional file 2: Table S1.** Histone peptides identified in the LC-MS analysis using *E. granulosus* and *B. taurus* database.

**Additional file 3: Figure S2.** Heat map of parasitic proteins identified in HF samples. All identified proteins are represented (blue: lower abundances; red: higher abundances), and their annotations are shown on the left.

**Additional file 4: Table S2.** Parasitic proteins identified by LC-MS in *E. granulosus* and *E. ortleppi* HF samples.

**Additional file 5: Table S3.** Comparative analysis of proteins identified by LC-MS in *E. granulosus* and *E. ortleppi* HF samples.

**Additional file 6: Table S4.** Predicted GO terms for proteins of unknown functions.

**Additional file 7: Table S5.** Bovine proteins identified by LC-MS in *E. granulosus* and *E. ortleppi* HF samples.

**Additional file 8: Figure S3.** Bovine proteins identified in hydatid fluid samples from pulmonary cystic echinococcosis. Venn diagrams showing the number of bovine proteins identified: **a** in *E. granulosus* HF samples; **b** in *E. ortleppi* HF samples; **c** in HF samples from each species or shared between them. The overall numbers of bovine proteins detected are indicated below the sample/species identification.

**Additional file 9: Table S6.** Comparative analysis of the proteins detected in at least two biological replicates in one of the species.

**Additional file 10: Table S7.** Common proteins from *E. granulosus* and *E. ortleppi* HF.

**Additional file 11: Table S8.** Functional classification and gene ontology (GO) enrichment analysis of proteins detected in hydatid fluid of *E. granulosus* and *E. ortleppi*.

**Additional file 12: Table S9.** Summarized GO categorization of proteins detected in *E. granulosus* hydatid fluid.

**Additional file 13: Table S10.** Summarized GO categorization of proteins detected in *E. ortleppi* hydatid fluid.

**Additional file 14: Table S11.** Gene ontology (GO) enrichment analysis of host proteins detected in hydatid fluid of *E. granulosus*.

**Additional file 15: Table S12.** Gene ontology (GO) enrichment analysis of host proteins detected in hydatid fluid of *E. ortleppi.*

## Data Availability

The datasets supporting the conclusions of this article are available in the ProteomeXchange Consortium repository, (http://proteomecentral.proteomexchange.org/cgi/GetDataset) with the dataset identifier PXD019314 and https://doi.org/10.6019/PXD019314.

## Abbreviations

AgB: Antigen B
Ag5: Antigen 5
CID: Collisional induced dissociation
Dhh: Desert Hedgehog Protein
EG: *Echinococcus granulosus*
ES: Excretory-secretory
EO: *Echinococcus ortleppi*
FDR: False discovery rate
GO: Gene ontology
HF: Hydatid fluid
HPLC: High performance liquid chromatography
HRM: High-resolution melting
LC/MS–MS: Liquid chromatography tandem mass spectrometry
NSAF: Normalized spectral abundance factor
PSC: Protoscoleces
SCX: Strong cation exchange
SDS-PAGE: Sodium dodecyl sulfate polyacrylamide gel electrophoresis
Wnt: Wingless/Integrated
