# Supplementary figures and images for: Proteomic profiling of hydatid fluid from pulmonary cystic echinococcosis
Source: Parasit Vectors. 2022 Mar 21;15:99. doi: 10.1186/s13071-022-05232-8 (PMC8935821; doi:10.1186/s13071-022-05232-8)

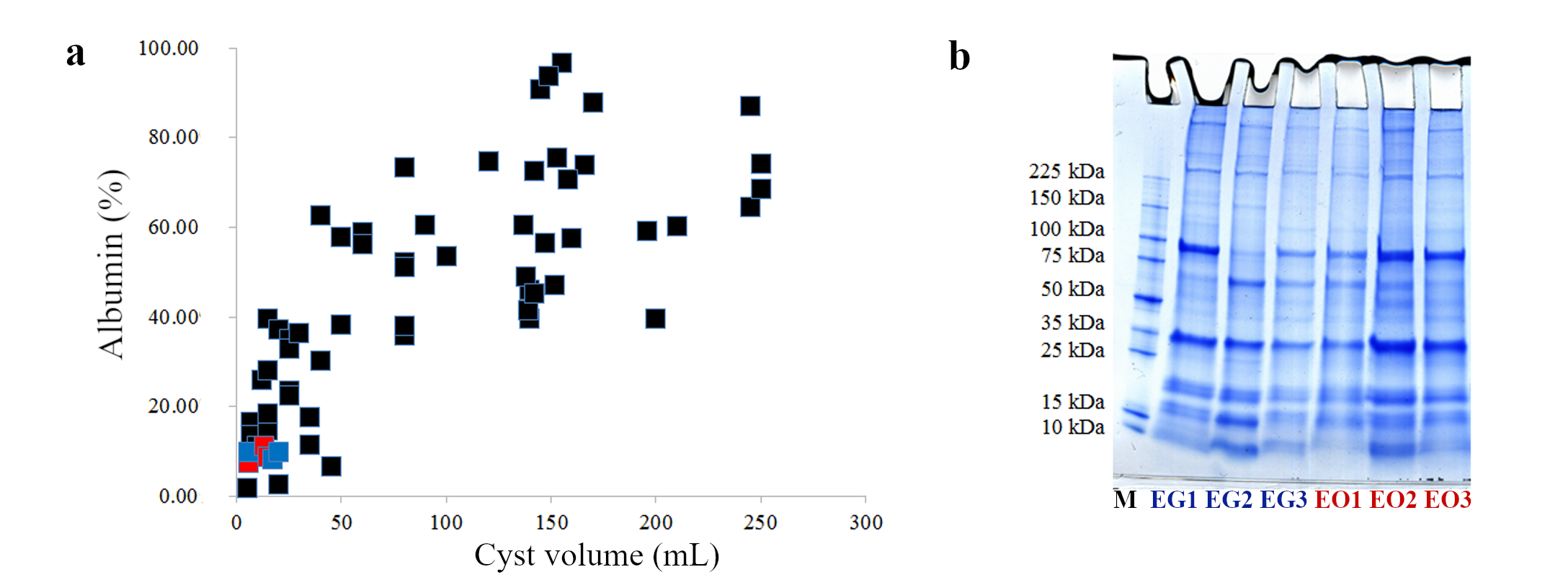

Supplement: Supplementary file 1 — Additional file 1: Figure S1. E. granulosus and E. ortleppi HF protein comparison. (A) Correlation between cysts volume and intensity of bovine albumin band. Thirty-four E. granulosus and 29 E. ortleppi HF samples were qualitatively evaluated using 12% SDS-PAGE gel. The intensity of the bovine albumin band, estimated by using IMAGEJ (https://imagej.nih.gov/ij/) to quantify band intensity, was correlated to the cyst volumes. The six HF samples from cysts with similar sizes (4–6 cm diameter) used in the proteomic analysis are indicated by blue squares (E. granulosus) and red squares (E. ortleppi). (B) Analysis of HF proteins from the selected samples. 50 μg of HF proteins E. granulosus (EG1–3) and E. ortleppi (EO1–3) samples were evaluated by 12% SDS-PAGE gel. For each sample it was possible to identify stained proteins from 10 to 250 kDa. Markers are indicated on the left. [file 13071_2022_5232_MOESM1_ESM.tif]

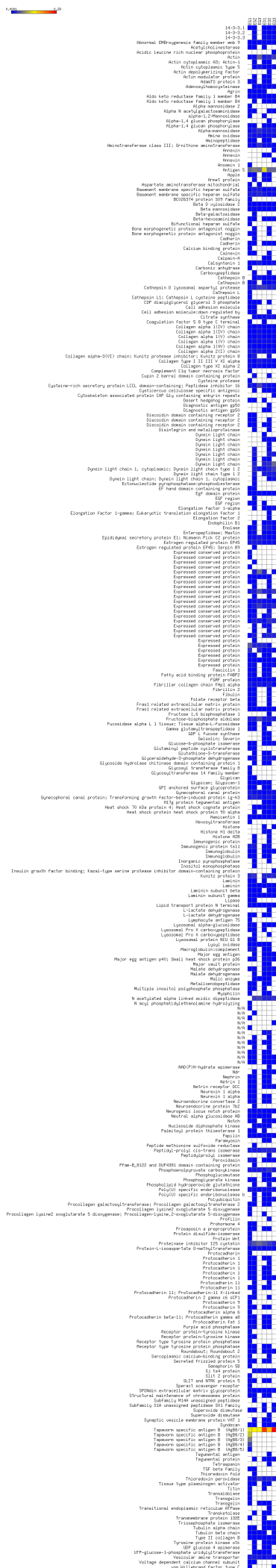

Supplement: Supplementary file 3 — Additional file 3: Figure S2. Heat map of parasitic proteins identified in HF samples. All identified proteins are represented (blue: lower abundances; red: higher abundances), and their annotations are shown on the left. [file 13071_2022_5232_MOESM3_ESM.pdf]

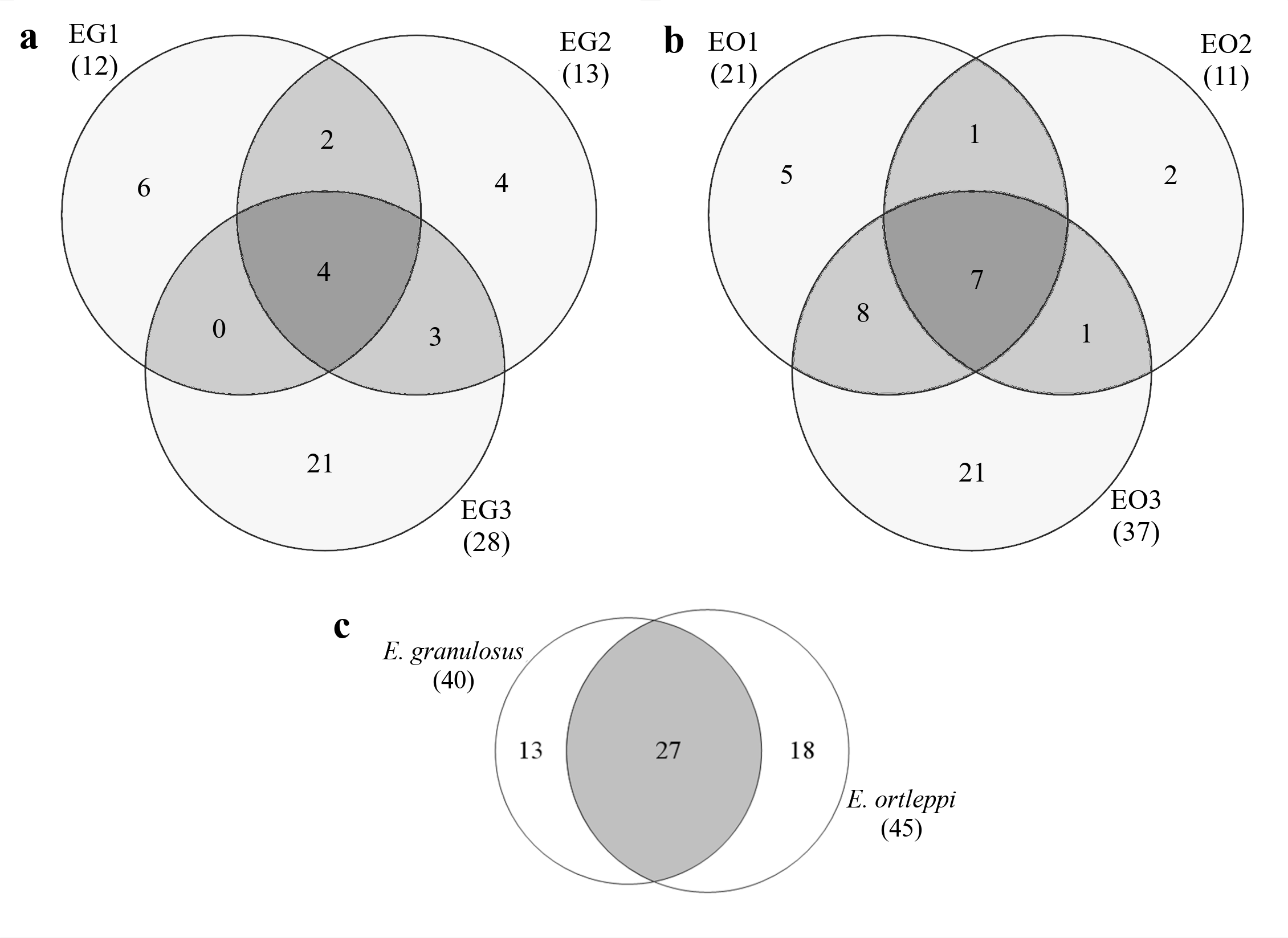

Supplement: Supplementary file 8 — Additional file 8: Figure S3. Bovine proteins identified in hydatid fluid samples from pulmonary cystic echinococcosis. Venn diagrams showing the number of bovine proteins identified: a in E. granulosus HF samples; b in E. ortleppi HF samples; c in HF samples from each species or shared between them. The overall numbers of bovine proteins detected are indicated below the sample/species identification. [file 13071_2022_5232_MOESM8_ESM.tiff]
